# Essential oil of *ocimum gratissimum* as a natural fungicide against pathogenic fungi of fruit crops and molecular docking studies

**DOI:** 10.1007/s42770-026-01964-2

**Published:** 2026-05-20

**Authors:** Armanda Aparecida Júlio, A. N. Venancio, G. R. de Souza, M. E. G. da Silva, L. A. Parreira, E. C. Aytar, M. S. Ferreira, H. O. G. Caprini, M. G. Soares, M. F. C. Santos, L. Menini

**Affiliations:** 1 Federal Institute of Espírito Santo, Alegre Campus, Espírito Santo Brazil; 2 Department of Chemistry and Phisical, Federal University of Espírito Santo, Alegre, Espírito Santo Brazil; 3 Agriculture Department of Horticulture, Usak University Faculty of Türkiye, Uşak, Türkiye Turkey; 4 Institute of Chemistry, Federal University of Alfenas, Alfenas, Minas Gerais Brazil; 5 Departament of Pharmacy and Nutrition, Federal University of Espírito Santo, Alegre, Espírito Santo Brazil

**Keywords:** *Ocimum gratissimum*, essential oil, fungal pathogens, molecular docking, biological activity

## Abstract

Fungal diseases in fruit crops compromise productivity even when synthetic fungicides are applied. However, growing health and environmental concerns have made such products less attractive to consumers. In this context, the present study aimed to extract and characterise the essential oil (EO) from leaves and flowers of African basil (*Ocimum gratissimum* L.) by hydrodistillation and to evaluate its bioactivity against the fungi *Botrytis cinerea*, *Fusarium guttiforme* and *Colletotrichum musae*. Average EO yields were 3.90% w/w (leaves) and 2.60% w/w (flowers). Chromatographic analysis identified four constituents, mainly mono- and sesquiterpenes, with eugenol as the major compound (> 80%). Both the EO and pure eugenol displayed fungicidal activity against *F. guttiforme* and *C. musae* at 0.50 and 0.80 µL mL⁻¹ (EO) and at 0.50 and 1.20 µL mL⁻¹ (eugenol), respectively. The EO and eugenol were fungistatic against *B. cinerea in vitro*. The EO also inhibited more than 95% of *C. musae* conidial germination from 0.20 µL mL⁻¹. In vivo assays, the EO reduced anthracnose severity in bananas. Molecular docking studies revealed that germacrene D and (E)-caryophyllene exhibited the strongest binding affinities to the fungal CYP51 enzyme, with binding energies of − 7.6 and − 7.3 kcal/mol, respectively, indicating their potential as effective antifungal agents. These findings indicate that African basil EO is a promising biofungicide and could be strategically integrated into sustainable crop protection programmes.

**Supplementary Information:**

The online version contains supplementary material available at 10.1007/s42770-026-01964-2.

## Introduction

Phytopathogens continue to challenge agriculture, impacting a wide range of crops. Among them, fungi are especially significant because they cause major economic losses. Globally, production losses attributed to fungal infections are estimated at up to 23% during pre-harvest and up to 20% post-harvest, even when fungicides are applied [1]. Fungal diseases diminish both the quantity and quality of agricultural products [2]. Fungi severely affect the fruit sector, which is vital for food and nutritional security because fruits contribute to a balanced and healthy diet. Post-harvest losses in fruit production may reach 55% of total yield [3].

Synthetic fungicides remain the primary strategy for controlling phytopathogenic fungi [4, 5]. Nevertheless, their use poses several risks, including human-health issues, environmental residue accumulation, the emergence of fungicide-resistant strains and threats to food safety [6, 7]. Consequently, alternative, more sustainable control methods are urgently needed.

Essential oils (EOs) have emerged as highly promising agents against diverse phytopathogenic fungi, and their antifungal activity is well documented [8–11]. Depending on the botanical source and concentration, EOs may exhibit fungicidal or fungistatic effects [12]. They are complex mixtures of volatile compounds found in various plant organs such as leaves, stems, roots, and fruits [13].

EOs have been tested against fungi that attack fruit crops, including species of *Botrytis*, *Fusarium* and *Colletotrichum*. For instance, oils from *Mentha spicata* and *Cymbopogon martini* effectively controlled *Botrytis cinerea*, reducing disease incidence in strawberries [14]. Recent studies likewise report that EOs from *Cymbopogon citratus*, *Origanum vulgare*, *Thymus vulgaris*, *Mentha aquatica*, *M. piperita*, *M. longifolia*, *Matricaria chamomilla*, *Pistacia atlantica*, conifers (*Chamaecyparis obtusa*, *C. pisifera* and *Thuja occidentalis*), *Syzygium aromaticum* and *Cinnamomum verum* are efficacious against *B. cinerea* [15–21].

*Fusarium* spp. infect a wide array of fruits and vegetables. Essential oils from *Cymbopogon winterianus*, *Zingiber officinale* and *Satureja hortensis* have shown promising control of these pathogens [22–24]. Regarding *Colletotrichum musae*, recent work highlights the antifungal efficacy of EOs from *Melaleuca alternifolia*, *Conyza bonariensis*, *Piper macedoi* Yunck., *Schleichera oleosa*, *Aniba canelilla* and *Aniba parviflora* [25–29].

Given this background, the present study aimed to extract and characterise the essential oil of African basil (*Ocimum gratissimum* L.) and to evaluate its fungicidal potential against three fungi of major importance to fruit production, *Fusarium guttiforme*, *Botrytis cinerea* and *Colletotrichum musae*, all of which are widely distributed among host species, causing significant economic losses and posing risks to food security.

## Materials and methods

### Extraction of essential oil and eugenol from ocimum gratissimum

The plant material, consisting of leaves and flowers of *O. gratissimum* (African basil), was collected in the morning from the Agronomy I sector of the Federal Institute of Espírito Santo – Alegre Campus, Alegre, Espírito Santo, Brazil (20° 45’ 50” S, 41° 27’ 15” W). The material was immediately separated, dried in a forced-air circulation oven at 40 °C (Marconi, MA033/630) until constant weight, and subsequently ground. The essential oil (EO) was obtained by hydrodistillation using a Clevenger-type apparatus. A plant material-to-water ratio of 0.8:10 was used, with a distillation time of 3 h. The hydrolate was collected using a micropipette and centrifuged (Hermle Labortechnik Centrifuge, Z 216 M) at 6000 rpm for 10 min. After centrifugation, the EO was transferred to amber glass tubes and stored in a freezer at -20 °C. EO yield was determined gravimetrically and expressed as the mean ± standard error of the mean. The eugenol used (99.99% purity) was purchased commercially from Sigma-Aldrich.

### Essential oil characterization

EO yield from *O. gratissimum* leaves and flowers was obtained from five independent extractions, with mean values calculated separately for each plant part. The identification of essential oil constituents was performed using chromatographic techniques. Mass spectra were compared to library data (Wiley Registry of Mass Spectral Data and NIST Mass Spectral Data), and calculated Retention Indices (IRcalc) were obtained using a standard solution of n-alkanes (C7–C30; Sigma-Aldrich). Retention indices were determined following Gonzalez and Nardillo [30] and compared with reference values from the literature [31]. Only compounds with relative peak area percentages ≥ 1% were included in the composition analysis.

EO samples were analysed by gas chromatography with flame ionisation detection (GC-FID, Shimadzu GC-2010 Plus) and gas chromatography–mass spectrometry (GC-MS, Shimadzu GC-MS-2010). Both analyses employed a fused-silica capillary column (30 m × 0.25 mm, 0.25 μm film thickness) with an SH-Rxi^®^-5HT stationary phase. Nitrogen was used as the carrier gas in GC-FID, and helium in GC-MS, with a flow rate of 3.0 mL/min. The oven program started at 40 °C (1 min hold), then ramped at 5 °C/min to 220 °C, held for 10 min. Injector and detector temperatures were maintained at 240 °C, using a split ratio of 1:30. For each run, 1.0 µL of a 2% (v/v) essential oil solution in ethanol (PA grade) was injected. GC-MS analysis employed electron impact ionisation (70 eV), scan speed of 0.50 fragments/s, and mass detection range from 29 to 400 m/z.

### Preparation of O. gratissimum essential oil formulations

Oil-in-water emulsions were prepared with 5% (v/v) essential oil, 2% (v/v) surfactant, and sterile distilled water. The emulsions were homogenised in an ultrasonic bath (42 kHz). Emulsion stability was tested based on surfactant type (Tween^®^ 20, Tween^®^ 80, Span^®^ 80, Span^®^ 85, soybean lecithin, or surfactant blends), stirring time (20, 30, 40, 50, or 60 min), and centrifugation (10 min at 5000 rpm). The most stable emulsion was achieved using a 1:1 Tween^®^ 80:Span^®^ 80 ratio and 50 min of agitation. These conditions were adopted to prepare the stock solutions for the biological assays.

### Fungal isolates

Strains of *B. cinerea* (B-68), *F. guttiforme* (CCF-449), and *C. musae* (CCF-243) were obtained from the Department of Plant Pathology at the Federal University of Viçosa (UFV), Minas Gerais, Brazil. These strains had been previously isolated from strawberry, pineapple, and banana, respectively.

### In vitro experiments on antifungal

In vitro tests were performed using the essential oil (a mixture of leaf and flower EOs) and pure eugenol, the EO’s major constituent. Two stock emulsions at 50 µL mL⁻¹ were prepared—one with only the essential oil and the other with pure eugenol. These stock solutions were diluted to obtain various concentrations for testing against the fungal pathogens. Preliminary trials were conducted to determine the working concentration range of the essential oil. Eugenol concentrations used in the assays matched those of the EO. The assays enabled estimation of the mycelial growth inhibitory concentration (IC₅₀) under laboratory conditions. EO treatments at different concentrations were added to Potato Dextrose Agar (PDA) medium, supplemented with amoxicillin to prevent opportunistic bacterial contamination. Once the medium solidified, 5 mm mycelial plugs (from 7-day-old fungal cultures) were placed at the centre of each plate. Plates were sealed with plastic film and incubated in a BOD chamber at 25 ± 1 °C, 12 h light/dark photoperiod, and 70 ± 10% relative humidity for 7 days, in a completely randomised design.

Commercial fungicides were used as positive controls: Cantus (1.50 µL mL⁻¹) for *B. cinerea*, Nativo (1.00 µL mL⁻¹) for *F. guttiforme*, and Tecto (0.92 µL mL⁻¹) for *C. musae*. As a negative control, an emulsion without essential oil or eugenol was applied. After incubation, the relative efficacy (RE) of each treatment was assessed by inhibition of mycelial growth using Eq. 1, adapted from Fiaccadori and Battistini [32]. Mycelial diameters were measured in two opposite directions with a digital caliper, and the mean values were used for calculations:

1 $$\:E.R.\:=\:\left(\frac{T\:-P}{T}\right)*100$$

Where P is the average mycelial growth in the treatment and T is the average growth in the negative control. The same procedure was used for testing pure eugenol.

### Fungicidal versus fungistatic action of the essential oil and eugenol

To determine whether *O. gratissimum* essential oil (EO) and pure eugenol act fungicidal or fungistatic, we used the minimum concentrations that produced 100% inhibition of mycelial growth in the previous assays. Five-millimetre mycelial plugs from the inhibited colonies were transferred to fresh Potato Dextrose Agar (PDA) and incubated for 7 days. Renewal of mycelial growth indicated a fungistatic effect, whereas the absence of growth denoted fungicidal activity. Because of the economic importance of the banana and its high susceptibility to anthracnose caused by *C. musae*, additional conidial-germination and in vivo fruit assays were carried out to assess the post-harvest efficacy of the EO.

### Influence of O. gratissimum EO on conidial germination

A conidial suspension of *C. musae* (1 × 10⁶ conidia mL⁻¹) was prepared. Aliquots of 130 µL were distributed onto 5-cm Petri dishes containing water-agar supplemented with 5% (v/v) EO emulsion at concentrations ranging from 0.04 to 0.80 µL mL⁻¹. The commercial fungicide Tecto^®^ (0.92 µL mL⁻¹) served as a positive control, and a 2% (v/v) surfactant blend (Tween 80 : Span 80, 1 : 1) acted as the negative control. Plates were incubated for 9 h at 25 °C in the dark. Afterwards, 100 conidia per replicate were examined microscopically (10×), and the germination-inhibition rate was calculated as (inhibited conidia / total conidia) × 100. The experiment comprised five replicates per treatment and followed the method of Zhang et al. [33], with minor adaptations.

### Effect of O. gratissimum EO on anthracnose in banana fruit

In vivo efficacy against *C. musae* was assessed using silver bananas (*Musa* spp.) according to Da Costa Gonçalves et al. [28]. Treatments were arranged in a completely randomised design: five EO concentrations (0.08, 0.20, 0.40, 0.60 and 0.80 µL mL⁻¹), a positive control (Tecto^®^, 0.92 µL mL⁻¹), and a negative control (distilled water).

Each treatment comprised three replicates, with three bananas per replicate. Fruits were sprayed with an airbrush sprayer (ON10, One Tools), placed on plastic trays lined with moistened filter paper (60 mL distilled water) and covered with sterile plastic containers. Incubation was at 25 ± 2 °C, 90% relative humidity, under a 12-h photoperiod. Disease progression, based on lesion appearance and expansion, was recorded at 8 days post-inoculation and monitored every two days thereafter.

### Molecular docking studies

In the molecular docking analyses conducted in this study, the target protein selected was CYP51 (lanosterol 14α-demethylase). CYP51 is a key enzyme in the ergosterol biosynthetic pathway in fungi and plays a vital role in maintaining the structural integrity and functionality of the fungal cell membrane. This enzyme is highly conserved across a wide range of pathogenic fungal species, making it an appropriate and widely accepted target for broad-spectrum antifungal studies. Furthermore, numerous reference molecules with known inhibitory activity against CYP51, along with crystallographically resolved enzyme structures, have been deposited in the Protein Data Bank (PDB). Accordingly, a structurally resolved CYP51 model—PDB ID: 5EQB, frequently used in computational studies, was selected as a representative structure for the in silico analyses. This approach enabled reliable, comparable evaluation of potential interactions relevant to general antifungal biological activity.

The selection of chemical compounds was based on the GC-MS and LTPRI characterization of *O. gratissimum* essential oil. Four major constituents, β-(cis)-cymene, eugenol, (E)-caryophyllene, and germacrene D, were identified and chosen for in silico evaluation based on their abundance and structural diversity.

Protein preparation was carried out using AutoDock Tools version 1.5.7. All crystallographic water molecules and co-crystallized ligands were removed, polar hydrogens were added, and Gasteiger partial charges were assigned to all atoms. The processed protein was then saved in PDBQT format for compatibility with AutoDock Vina. Ligand structures were manually drawn in ChemDraw and subsequently converted into three-dimensional conformations. These were imported into AutoDock Tools, where torsional bonds were defined and atomic charges were calculated. All ligands were saved in PDBQT format.

Docking simulations were performed using AutoDock Vina version 1.2.0. For compounds derived from *Ocimum gratissimum*, the docking grid was centered at X = − 38, Y = − 14, Z = 25 to target the active site of the CYP51 enzyme. The grid box dimensions were adjusted to fully encompass the binding pocket, and the exhaustiveness parameter was set to 8 to ensure sufficient conformational sampling. Following docking, the resulting binding affinities (kcal/mol) and binding poses were analyzed. Key molecular interactions, including hydrogen bonds, hydrophobic contacts, and π–π stacking interactions, were visualized and interpreted using Discovery Studio Visualizer 2021.

### Statistical analysis

Data from the in vitro experiments were analysed using R^®^ software version 3.4.4 (R Core Team, 2018). Results were subjected to analysis of variance (ANOVA) at a 5% significance level.

## Results and discussion

### Phytochemical composition of ocimum gratissimum essential oil

The essential oils (EOs) extracted separately from the leaves and flowers of *O.gratissimum* showed average yields of 3.90 ± 0.17% w/w (leaves) and 2.61 ± 0.42% w/w (flowers). Chromatographic analyses revealed that the EOs obtained from both plant parts shared the same chemical composition and in equivalent proportions. Therefore, the leaf and flower oils were combined to form a single EO, which was used throughout this study. The mixed essential oil of *O. gratissimum* was composed of four major compounds. Only constituents with a relative area greater than 1%, based on peak normalization and adjusted using relative response factor simulation, were considered in the final composition. Chromatographic analysis led to the identification of these four primary components, which are listed in Table 1 and illustrated in Figure S1 (Supplementary Material).

**Table 1:**
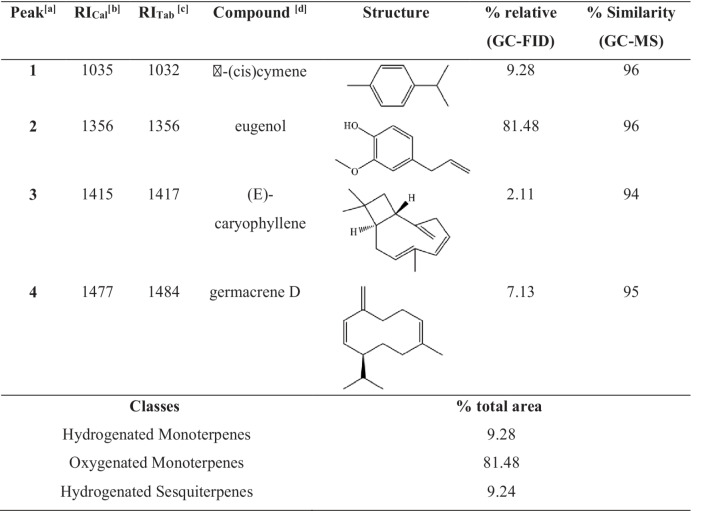
Characterization by the LTPRI index and GC-MS of the essential oil of basil flowers and leaves

^[a]^ Peaks based on chromatogram in figure S1 of Supplementary materials, compounds with relative areas >1% were identified. ^[b]^ RI_Cal_ (LTPRI): Retention Index calculated through data obtained by sample of saturated n-alkanes (C7-C30). ^[c]^ RI_Tab:_ Tabulated Retention Index on Adams (2007). ^[d]^ Compounds were identified by retention index (GC-FID) and mass spectrometry (GC-MS) using Rtx®-5MS column.

Essential oils are primarily composed of mono- and sesquiterpenes, which may or may not contain oxygen in their structure [34, 35]. The EO from *O. gratissimum*, a species in the Lamiaceae family and widely known for its aromatic properties, is dominated by monoterpenes. Among these, eugenol was identified as the major constituent, accounting for over 80% of the total composition. The predominance of mono- and sesquiterpenes in essential oils is associated with their volatility and biosynthetic origin. The specific composition of EO constituents is largely influenced by the regulation of biosynthetic pathways, which can vary in response to the plant’s physiological demands [36, 37].

### In vitro experiments antifungal

**Fig. 1:**
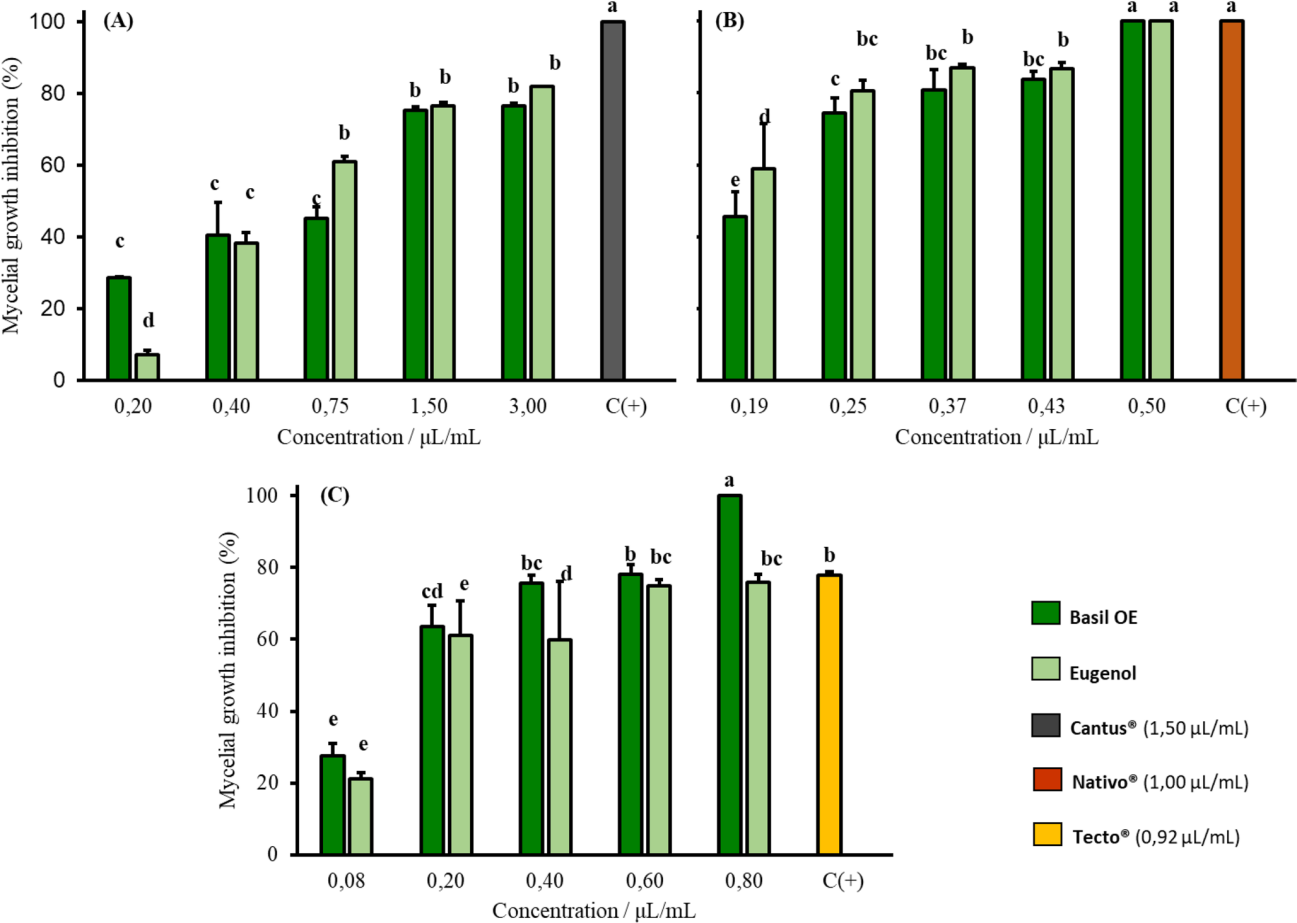
Relative efficiency of basil EO and eugenol in inhibiting mycelial growth of *B. cinerea* (**A**), *F. guttiforme* (**B**) and *C. musae* (**C**). ^a^Mycelial inhibition average ± standard deviation. Identical letters in the column indicate no significant difference (Tukey’s test, p-value < 0.05)

The dose-dependent effects of *O. gratissimum* essential oil (EO) and eugenol on mycelial growth varied depending on the fungal species evaluated (*B. cinerea*, *F. guttiforme*, and *C. musae*). Lower concentrations of both EO and eugenol were sufficient to inhibit *F. guttiforme*, while higher concentrations were required to inhibit *B. cinerea* (Fig. 1).

Both *O. gratissimum* EO and its main compound, eugenol, were effective in suppressing the mycelial growth of *B. cinerea*, *F. guttiforme*, and *C. musae* under in vitro conditions. These results suggest that both the EO and eugenol have promising antifungal activity against pathogens from three distinct genera. Experimental data are presented in Figures S2, S3, S4 and S5 (Supplementary Material). In assays against *B. cinerea* (Fig. 1A), neither the EO nor eugenol achieved complete inhibition at concentrations equal to or greater than 1.50 µL/mL. However, both treatments reduced fungal growth by nearly 80%, indicating good potential for managing this pathogen.

*O. gratissimum* EO was previously evaluated by Tripathi, Dubey, and Shukla [38] against *B. cinerea* in grapes, with maximum fungitoxic activity reported at 500 mg/mL. However, that study did not mention eugenol nor provide a chemical profile of the EO used. In a more recent study by De Albuquerque Sousa [21], the antifungal activity of eight different EOs against *B. cinerea* was assessed. Cinnamon, clove, and lemon verbena oils showed the strongest activity, with their main active compounds identified as cinnamaldehyde, eugenol, β-citral, and citronellal.

For *F. guttiforme* (Fig. 1B), the EO of *O. gratissimum*, at a concentration 50% lower (0.50 µL/mL) than that of the commercial fungicide (positive control), demonstrated greater inhibitory activity than the chemical product commonly used in pineapple cultivation. This effect may be attributed to synergism among the EO constituents. Eugenol, at 0.60 µL/mL, exhibited an inhibitory effect comparable to that of the positive control.

In tests against *C. musae* (Fig. 1C), both the EO and eugenol were effective at 0.80 µL/mL, a concentration lower than that of the commercial fungicide used as positive control. These results indicate strong potential for *O. gratissimum* EO in controlling *C. musae*, the causative agent of anthracnose in bananas. A study by Madjouko et al. [39] reported total inhibition of *C. musae* mycelial growth using *O. gratissimum* EO at just 0.275 µL/mL, lower than the values observed in the present study. However, the chemical composition of that study differed significantly: thymol (~ 43%) was identified as the major compound, whereas eugenol (~ 81%) was the dominant compound in our EO. This explains the disparity in efficacy between studies using oils from the same plant species.

Differences in EO composition and yield can be explained by the findings of Aboukhalid et al. [40], who showed that environmental factors (edaphoclimatic conditions) and plant genetics strongly influence the chemical profile of EOs, even within the same species. Therefore, variations in the composition and concentrations of active constituents are expected in EO studies.

For all three phytopathogens tested in this study, *O. gratissimum* EO and eugenol demonstrated strong potential for fungal control, especially in the postharvest phase when fruit losses are most critical. Compared to synthetic fungicides, EOs offer multiple advantages: they consist of biodegradable and volatile compounds, leave little to no residue, and pose minimal environmental risk [41]. The results of the fungicidal and fungistatic actions of *O. gratissimum* EO and eugenol against the three fungi tested, along with the estimated IC₅₀ values, are shown in Table 2. The graphs used to estimate these values are presented in Figures S6, S7, and S8 (Supplementary Material).

**Table 2:** Action of basil EO and eugenol against the fungi *B. cinerea*, *F. guttiforme* and *C. musae*, as well as the IC_50_ estimates for each experiment

Fungi	EO		Eugenol		IC_50_ *EO	IC_50_ *Eugenol
	[fungicide]*	fungistatic	[fungicide]*	fungistatic		
*B. cinerea*	-	Sim	-	Sim	0.70	0.65
*F. guttiforme*	0.50	-	0.50	-	0.18	0.13
*C. musae*	0.80	-	1.20	-	0.15	0.24

*Concentration unit: µL/mL

Both the *O. gratissimum* essential oil (EO) and eugenol displayed fungistatic activity against *B. cinerea* at all tested concentrations yet acted fungicidally against *F. guttiforme* and *C. musae*. For *F. guttiforme*, the minimum fungicidal concentration (MFC) was identical for both products, 0.50 µL mL⁻¹. In the case of *C. musae*, the EO required only 0.80 µL mL⁻¹ to reach the MFC, whereas eugenol alone needed 1.20 µL mL⁻¹, higher than the commercial fungicide benchmark.

### Influence of the essential oil on C. musae conidial germination

**Fig. 2:**
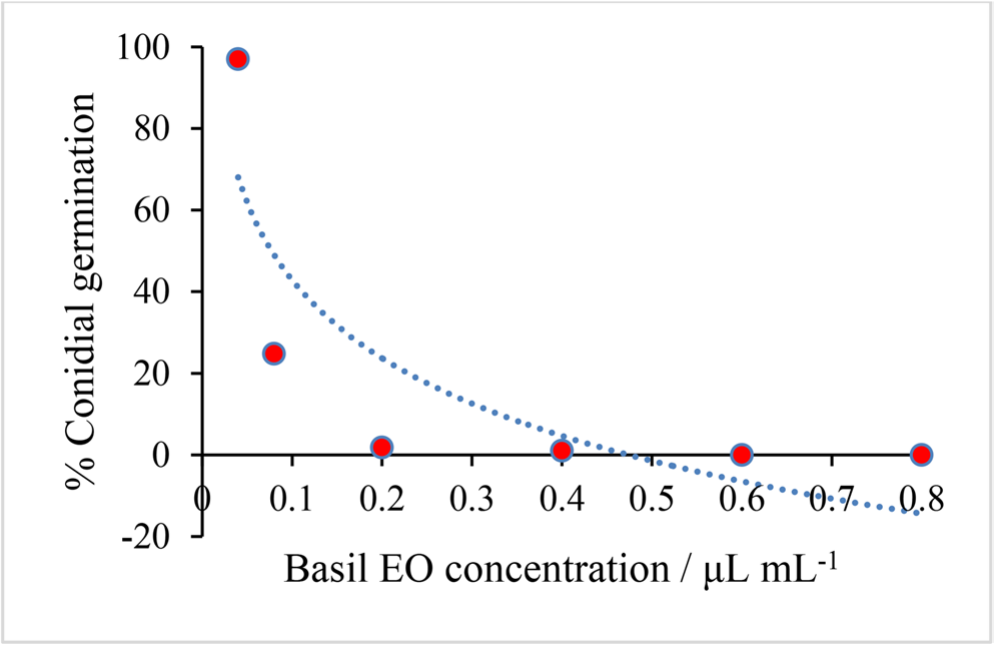
Tendency of basil EO to inhibit the germination of *C. musae conidia*

The EO markedly suppressed *C. musae* conidial germination: inhibition exceeded 95% from 0.20 µL mL⁻¹ onward (Fig. 2; Figure S9, Supplementary Material). These findings confirm that the oil not only curbs mycelial growth but also blocks the formation of infectious propagules, underscoring its value as a fungicide.

Madjouko et al. [39] likewise reported complete inhibition of *C. musae* conidia by *O. gratissimum* EO. Preventing or delaying germination is critical because once a conidium germinates, it quickly differentiates penetration structures that invade host tissues, induce necrosis, and lead to post-harvest losses [42].

### Effect of O. gratissimum EO on banana anthracnose

Disease-severity assessments eight days after treatment showed a clear, dose-dependent decline in anthracnose lesions on ‘Prata’ bananas. Higher EO concentrations produced smaller necrotic areas, indicating strong fungicidal activity. Across all doses, the EO outperformed the commercial fungicide (positive control) while offering the added benefits of biodegradability and low environmental persistence. Curiously, the lowest EO dose (0.08 µL mL⁻¹) appeared to favour lesion development, possibly because uneven ripening among fruit introduced additional variability. Representative photographs of the in vivo assay are provided in Figure S10 (Supplementary Material).

### Result of molecular docking studies

**Fig. 3:**
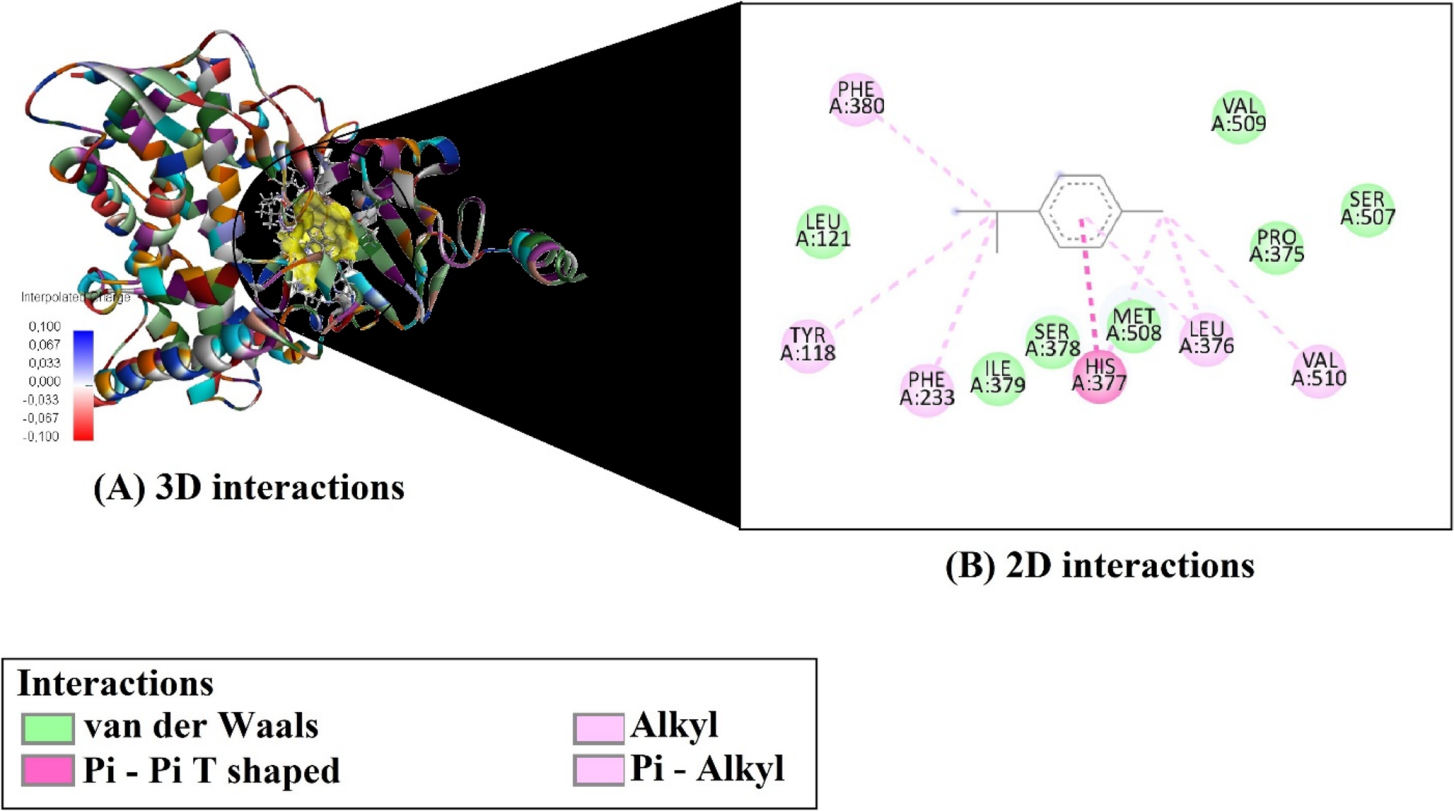
3D and 2D interaction analysis of Germacrene D with the CYP51 enzyme active site

**Fig. 4:**
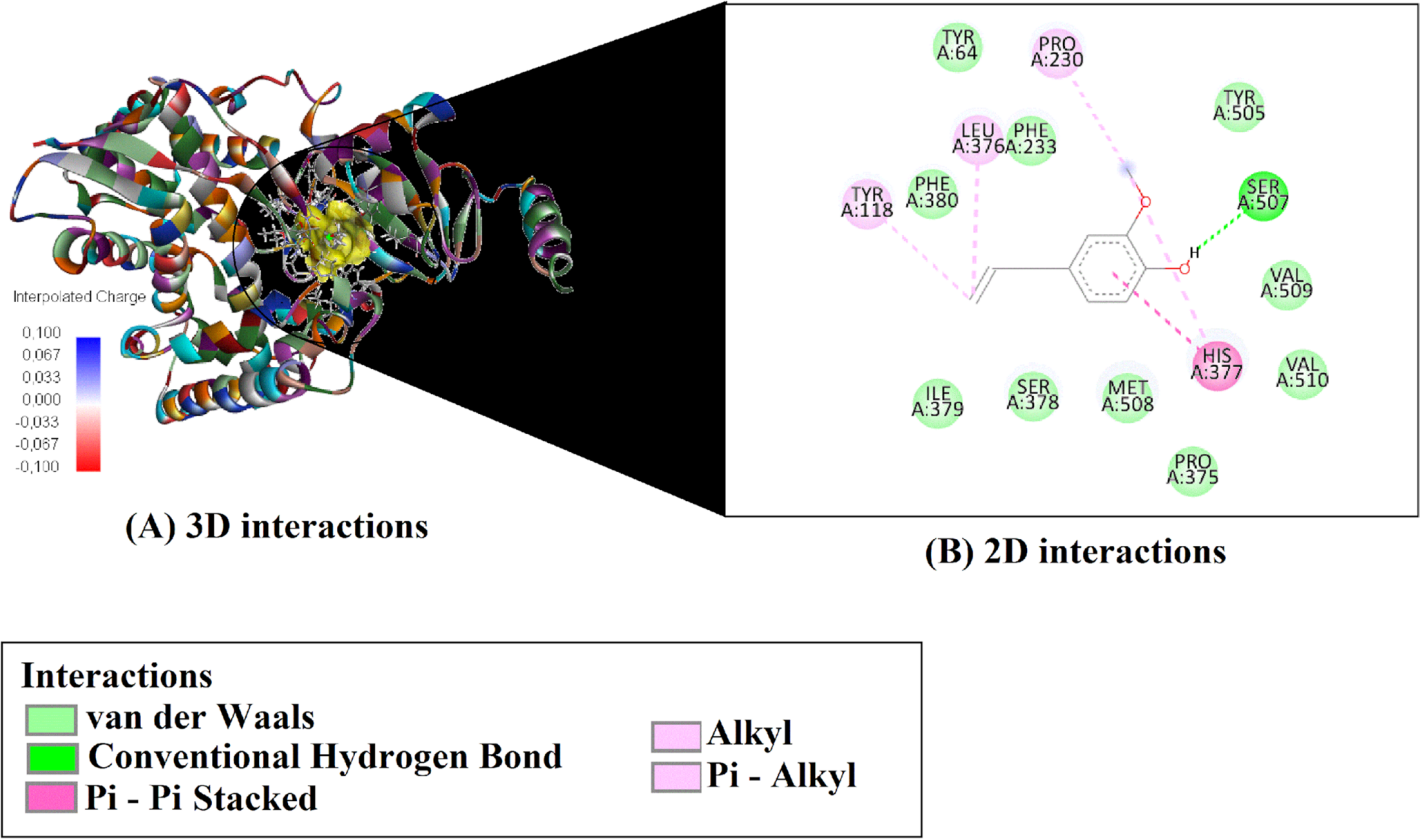
3D and 2D interaction analysis of (E)-caryophyllene with the CYP51 enzyme active site

**Fig. 5:**
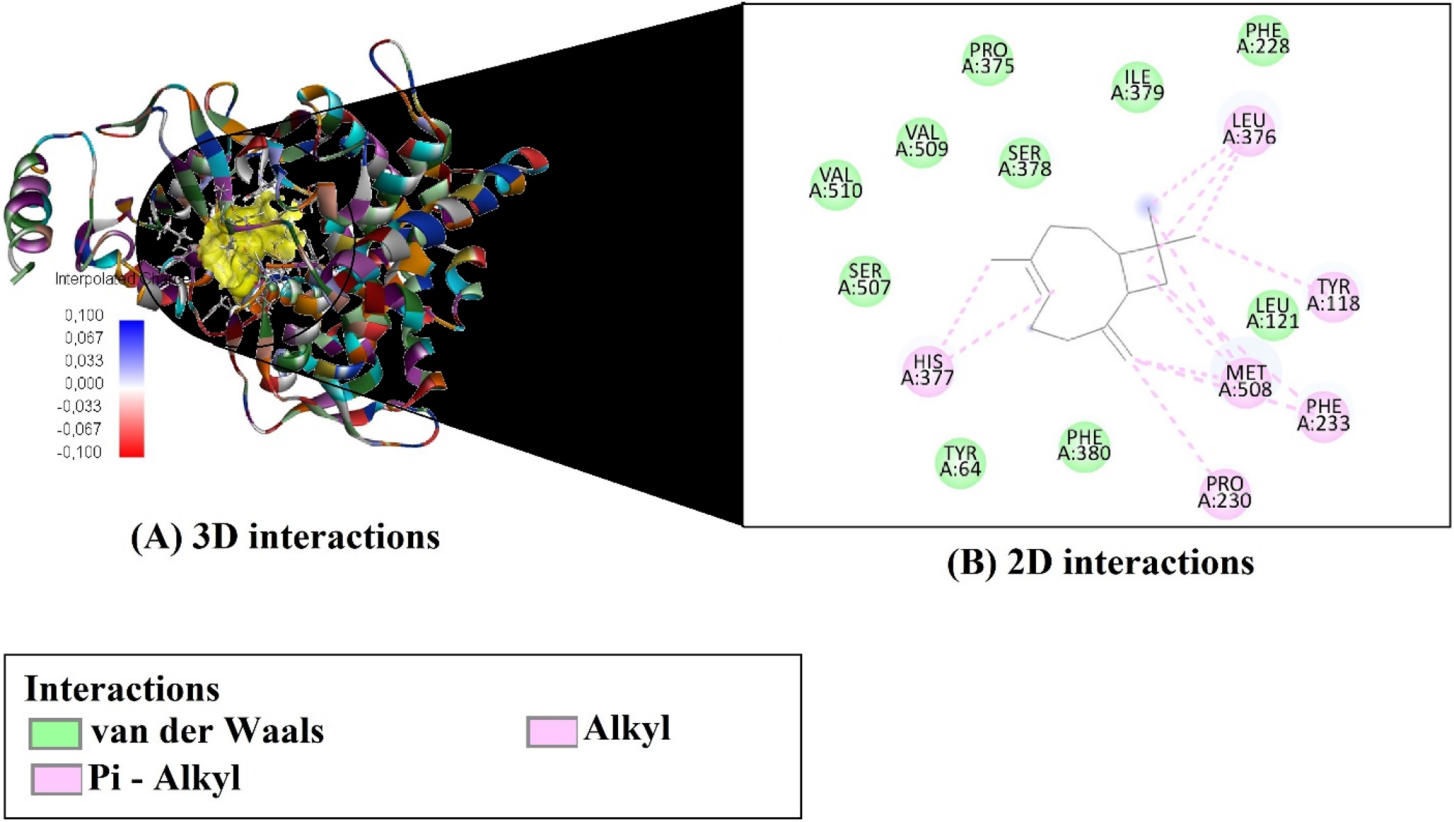
3D and 2D interaction analysis of beta-(cis)-cymene with the CYP51 enzyme active site

**Fig. 6:**
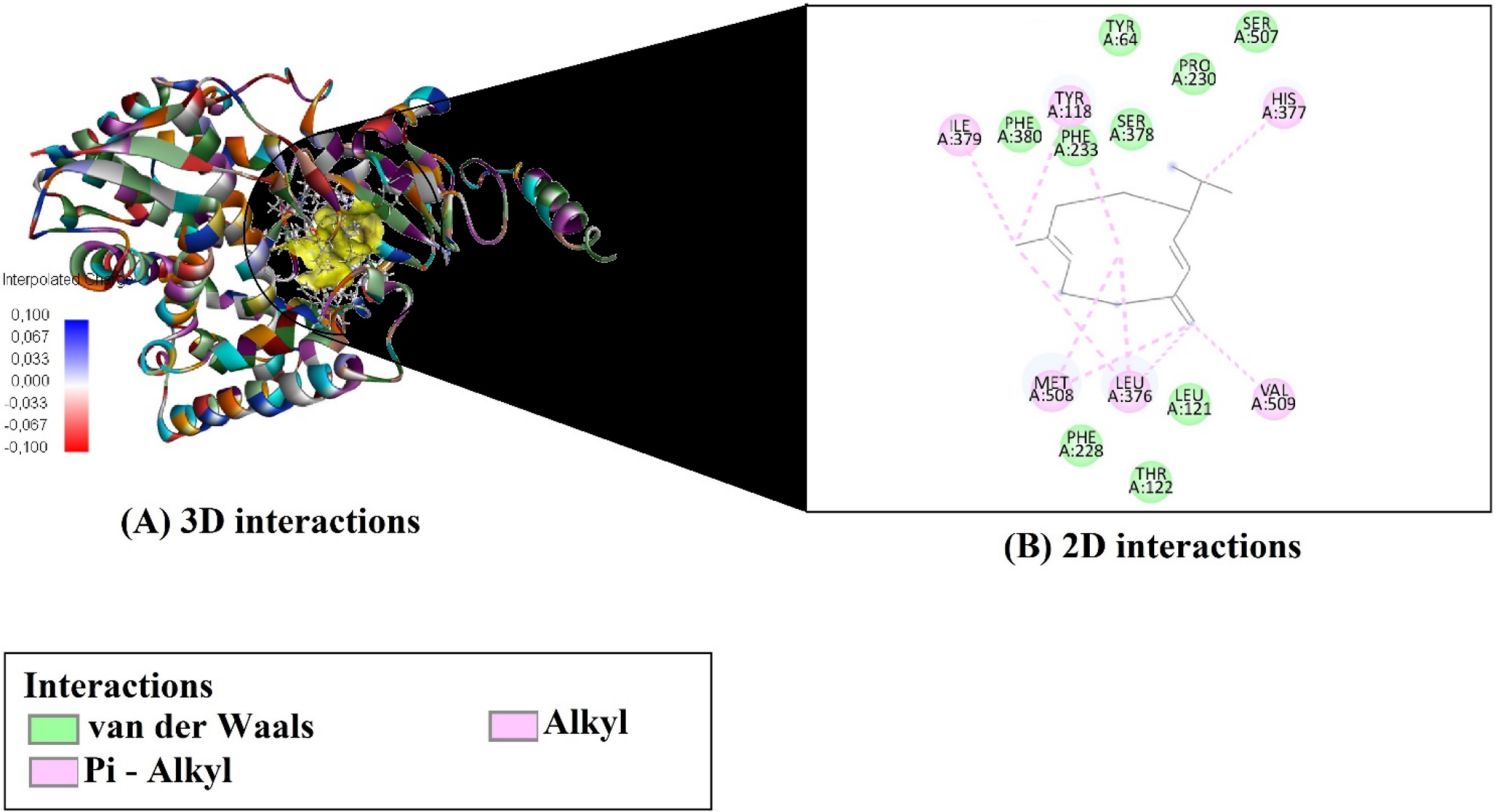
3D and 2D interaction analysis of Eugenol with the CYP51 enzyme active site

Based on molecular docking simulations, the binding affinities and efficiency metrics of four major compounds from *O. gratissimum* essential oil against the fungal target enzyme CYP51 (PDB ID: 5EQB) are presented in Table 3. Among the evaluated compounds, Germacrene D exhibited the strongest binding affinity (–7.6 kcal/mol) (Fig. 3), followed by (E)-caryophyllene (–7.3 kcal/mol) (Fig. 4), β-(cis)-cymene (–6.4 kcal/mol) (Fig. 5), and Eugenol (–5.9 kcal/mol) (Fig. 6). In terms of ligand efficiency (LE), the highest value was observed for β-(cis)-cymene (0.640), indicating a strong binding contribution per heavy atom. This compound also demonstrated the highest Fit Quality (FQ: 1.041) and Binding Efficiency Index (BEI: 0.048), suggesting a favorable physicochemical balance. However, Germacrene D showed the lowest estimated inhibition constant (Ki: 2.550 µM), indicating the greatest inhibitory potential among the tested molecules. Collectively, these findings suggest that Germacrene D and (E)-caryophyllene are the most promising antifungal candidates due to their strong binding affinities and low Ki values, while β-(cis)-cymene demonstrates notable efficiency metrics despite a slightly weaker binding energy.

**Table 3:** Docking-based binding affinities and efficiency-related parameters of major compounds from *Ocimum gratissimum* essential oil against CYP51 (PDB ID: 5EQB)

beta -(cis)-cymene	Binding Energy (kcal/mol)	LE	FQ	BEI	Ki (µM)
	-6.4	0.640	1.041	0.048	22.438
eugenol	-5.9	0.590	0.933	0.036	51.137
(E)-caryophyllene	-7.3	0.487	0.821	0.036	4.036
germacrene D	-7.6	0.507	0.855	0.037	2.550

* *BEI* Binding Efficiency Index, *FQ* Fit Quality, *Ki* Estimated Inhibition Constant, *LE* Ligand Efficiency,

The molecular interaction profiles of the four major compounds from *O. gratissimum* essential oil with the active site residues of the CYP51 enzyme (PDB ID: 5EQB) are summarized in Table 4. Among the analyzed ligands, β-(cis)-cymene formed a π–π T-shaped interaction with HIS377 and established several alkyl and π–alkyl interactions with residues such as LEU376, VAL510, TYR118, PHE233, and PHE380. Eugenol was the only compound to form a conventional hydrogen bond with SER507 and to engage in π–π stacking with HIS377, as well as multiple hydrophobic interactions with LEU376, PRO230, and TYR118.

(E)-caryophyllene and germacrene D did not display any hydrogen bonding or π–π stacking, but both exhibited extensive hydrophobic interactions within the binding site. (E)-caryophyllene interacted via multiple alkyl and π–alkyl contacts, particularly with LEU376, MET508, and PHE233, while germacrene D showed similar binding through contacts with LEU376, VAL509, ILE379, and HIS377. These findings highlight the importance of hydrophobic interactions, especially alkyl and π–alkyl contacts, in stabilizing ligand binding within the CYP51 active site.

**Table 4:** Key molecular interactions between major compounds from *O. gratissimum* essential oil and the active site residues of CYP51

Compounds	H-Bond	π-Stacking / π-Electrostatic	Alkyl / π-Alkyl Interactions
beta -(cis)-cymene	-	HIS377 (Pi–Pi T-shaped)	LEU376 (Alkyl + Pi–Alkyl), VAL510 (Alkyl), TYR118 (Pi–Alkyl), PHE233 (Pi–Alkyl), HIS377 (Pi–Alkyl), PHE380 (Pi–Alkyl)
eugenol	SER507–H6(Conventional)	HIS377 (Pi–Pi Stacked)	LEU376 (Alkyl), PRO230 (Alkyl), TYR118 (Pi–Alkyl), HIS377 (Pi–Alkyl)
(E)-caryophyllene	-	-	LEU376 (Alkyl ×3), MET508 (Alkyl ×3), PRO230 (Alkyl), TYR118 (Pi–Alkyl), PHE233 (Pi–Alkyl ×2), HIS377 (Pi–Alkyl ×2)
germacrene D	-	-	LEU376 (Alkyl ×3), MET508 (Alkyl ×2), VAL509 (Alkyl), ILE379 (Alkyl), TYR118 (Pi–Alkyl ×2), HIS377 (Pi–Alkyl)

Fungal infections have emerged as a serious global public health concern, accounting for approximately 13 million cases and 1.5 million deaths worldwide [43–45]. Depending on factors such as the fungal species involved, the duration of infection, and the individual’s overall health, these infections can range from asymptomatic to life-threatening [46]. In particular, immunocompromised individuals, as well as patients with impaired skin or mucosal integrity due to burns, wounds, or invasive medical interventions, are more susceptible to opportunistic fungal infections [47–49].

Lanosterol 14α-demethylase (CYP51) is an enzyme that belongs to the cytochrome P450 family. CYP51 plays a key role in ergosterol biosynthesis by catalyzing the oxidative removal of the 14α-methyl group from lanosterol and 24 [28]-methylene-24,25-dihydrolanosterol [50]. This enzyme is critical to the fungal life cycle and is a major target in antifungal drug design. Specific inhibition of CYP51 leads to a reduction in ergosterol, a structural component of the fungal cell membrane, resulting in the intracellular accumulation of lanosterol and other methylated sterols, thereby halting fungal cell growth [51].

Azole compounds (those containing imidazole, triazole, or tetrazole groups) are widely used in the treatment of fungal infections due to their mechanism of action, which targets the CYP51 enzyme, and their bacteriostatic effects. However, despite their effectiveness, the emergence of resistant fungal strains in recent years has posed a significant challenge in clinical practice. This growing resistance has shifted research efforts towards the design and development of non-azole inhibitors targeting CYP51 [50]. In our study, the antifungal potential of β-(cis)-cymene, eugenol, (E)-caryophyllene, and germacrene D, compounds targeting CYP51, was comprehensively analyzed using molecular modeling approaches.

It can be suggested that β-(cis)-cymene, eugenol, (E)-caryophyllene, and germacrene D show significant potential to bind to the active site of CYP51 and exert antifungal effects. This interaction may explain why (E)-caryophyllene and germacrene D (with binding energies of − 7.3 and − 7.6 kcal/mol, respectively) exhibit stronger binding affinities and greater antifungal activity than the other compounds.

In the study by Zarei et al. [52], dehydroabietic acid (DAA), 3-hydroxy-2,2-dimethyl-8-prenylchromane-6-propenoic acid (3HPP), and (2R,3R)-6[1-(4′-hydroxy-3′-methoxyphenyl)] (HYM) were reported to bind to the lanosterol 14α-demethylase enzyme with binding energies of − 10.4, − 10.0, and − 9.8 kcal/mol, respectively, indicating strong affinity. Among these, DAA and 3HPP were identified as the most promising antifungal agent candidates targeting CYP51. Similarly, our study suggests that caryophyllene and germacrene D may act by targeting the lanosterol biosynthetic pathway and thus could serve as promising new antifungal agents with potential bacteriostatic properties.

## Conclusion

The essential oil of *O. gratissimum*, rich in eugenol (> 80%), exhibited pronounced antifungal activity. It inhibited ~ 80% of *B. cinerea* mycelial growth at 3.00 µL mL⁻¹ and achieved complete inhibition of *F. guttiforme* and *C. musae* at 0.50 and 0.80 µL mL⁻¹, respectively. Germination of *C. musae* conidia was completely suppressed from 0.60 µL mL⁻¹ upward. In vivo tests confirmed its efficacy against banana anthracnose, extending shelf life and outperforming a commercial fungicide. While eugenol alone was more potent against *F. guttiforme* (MFC = 0.43 µL mL⁻¹), the complete oil was superior against *C. musae*, suggesting synergistic interactions among its constituents. Germacrene D and (E)-caryophyllene, with strong binding affinities to the CYP51 enzyme, emerged as the most promising antifungal candidates in molecular docking analyses. Altogether, these results position *O. gratissimum* EO as a promising, eco-friendly alternative for managing fungal diseases in agriculture.

## Supplementary information

Below is the link to the electronic supplementary material.

Supplementary Material 1
