# Supplementary material for: Essential oil of ocimum gratissimum as a natural fungicide against pathogenic fungi of fruit crops and molecular docking studies
Source: Braz J Microbiol. 2026 May 20;57(1):151. doi: 10.1007/s42770-026-01964-2 (PMC13190914; doi:10.1007/s42770-026-01964-2)
Supplement: Supplementary file 1 — Supplementary Material 1 [file 42770_2026_1964_MOESM1_ESM.docx]

Essential oil *of Ocimum gratissimum* as a natural fungicide against pathogenic fungi of fruit crops and molecular docking studies

Armanda Aparecida Júlio^a^, A. N. Venancio^a^, G. R. de Souza^a^, M. E. G. da Silva^a^, M. A. B. Silva^a^, L. A. Parreira^b^, E. C. Aytar^c^, M. S. Ferreira^d^, H. O. G. Caprini^e^, M. G. Soares^d^, M. F. C. Santos^b^*, L. Menini^a^

^a^Federal Institute of Espírito Santo, Alegre Campus, Alegre – Espírito Santo, Brazil; ^b^Department of Chemistry and Phisical, Federal University of Espírito Santo, Alegre – Espírito Santo, Brazil; ^c^Agriculture Department of Horticulture, Usak University Faculty of Türkiye, Uşak, Türkiye; ^d^Institute of Chemistry, Federal University of Alfenas, Alfenas – Minas Gerais, Brazil.; ^e^Departament of Pharmacy and Nutrition, Federal University of Espírito Santo, Alegre - Espírito Santo, Brazil

*[mariosantos408@gmail.com](mailto:mariosantos408@gmail.com)

**SUPPLEMENTARY MATERIAL**

**3**

**1**

**2**

**4**

**Figure S1.** Chromatographic profile of essential oil obtained from basil flowers and leaves.


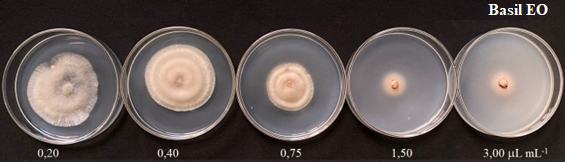


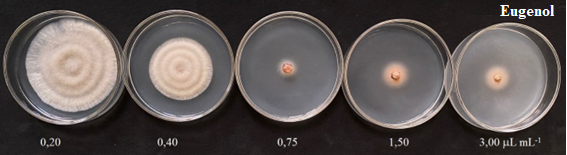


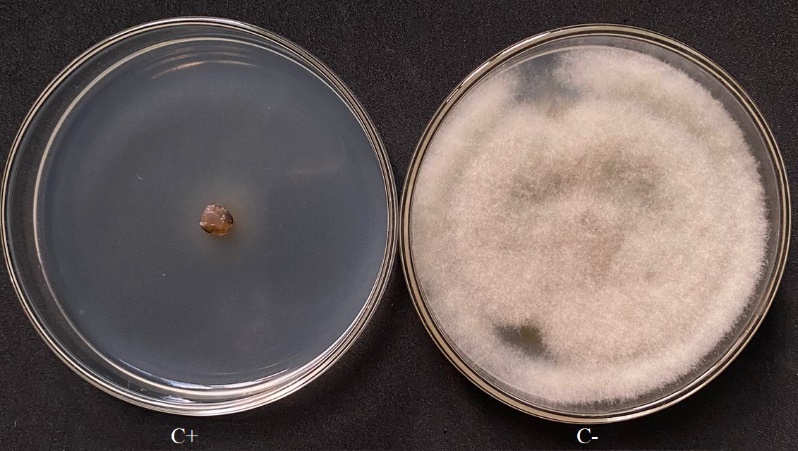


**Figure S2.** Photographs of the experimental results of evaluating the action of basil EO and eugenol against *Botrytis cinerea*.


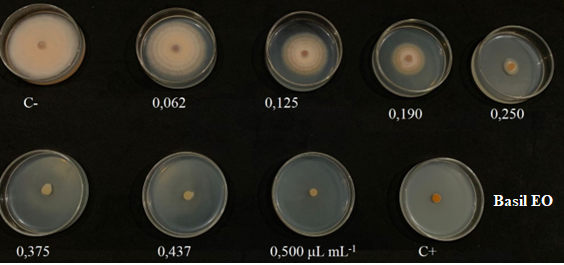


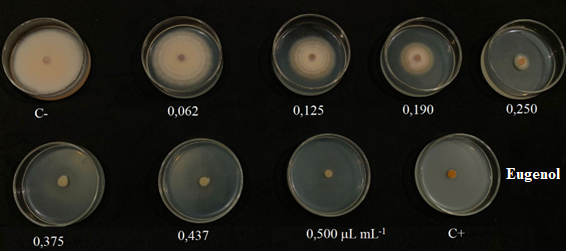


**Figure S3.** Photographs of the experimental results of evaluating the action of basil EO and eugenol against *Fusarium guttiforme*.


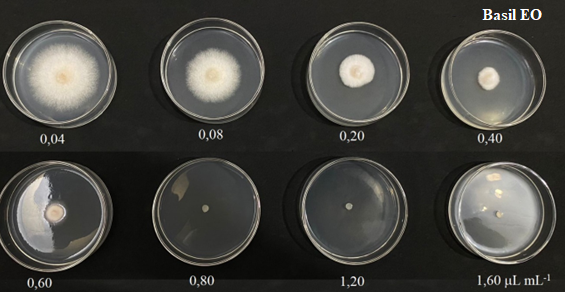


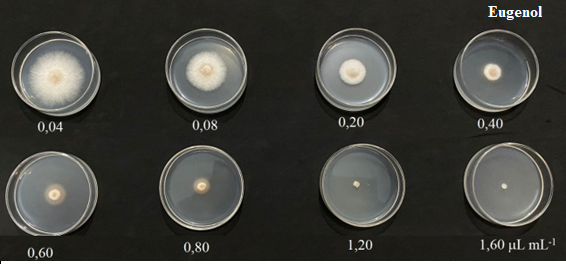


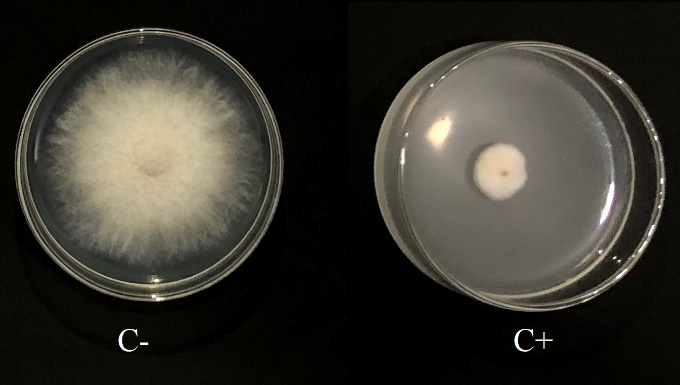


**Figure S4.** Photographs of the experimental results of evaluating of the action of basil EO and eugenol against *Colletotrichum musae*.

**Figure S5.** Graphs used to estimate IC_50_ against mycelial growth of *B. cinerea* using basil EO and eugenol, respectively.

**Figure S6.** Graphs used to estimate IC_50_ against mycelial growth of *F. guttiforme* using basil EO and eugenol, respectively.

**Figure S7.** Graphs used to estimate IC_50_ against mycelial growth of *C. musae* using basil EO and eugenol, respectively.


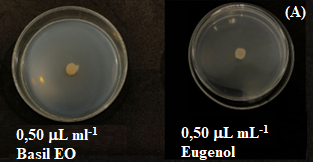


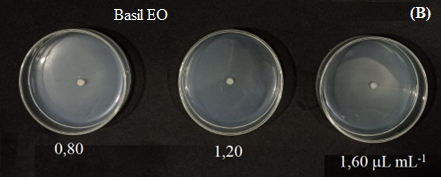


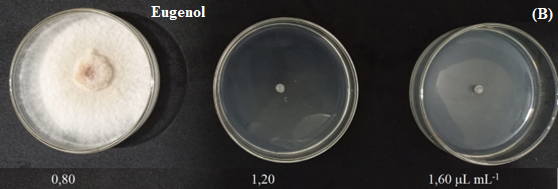


**Figure S8.** Photographs of the fungicidal assay with basil essential oil and eugenol against the fungi F. guttiforme (A) and C. musae (B), obtained after the incubation of plates in a new culture medium.


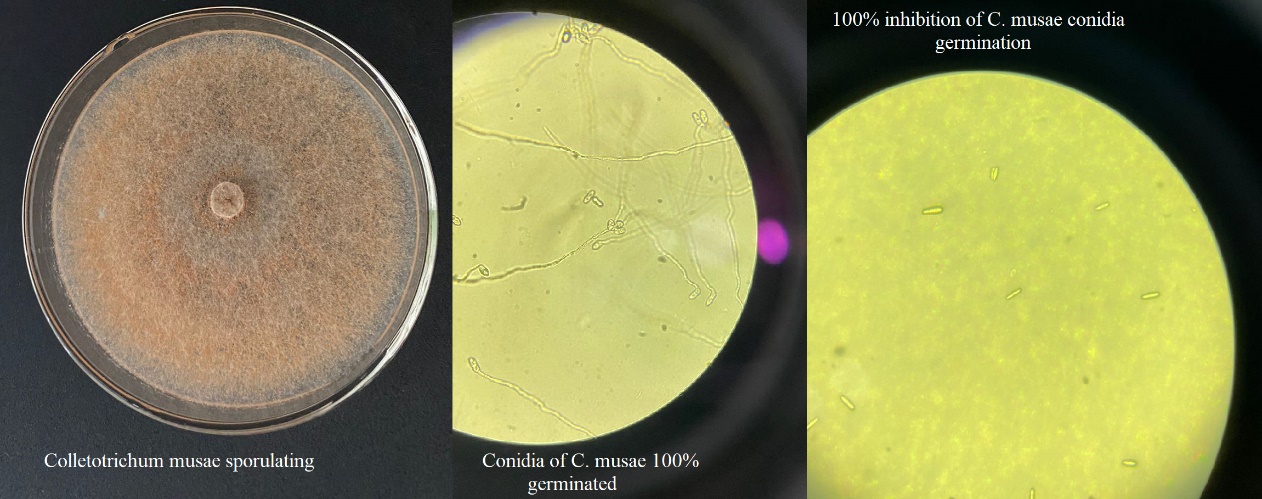


**Figure S9.** It experimentally obtained photographs showing the fungus *C. musae* sporulating, when it is 100% germinated and when the action of basil EO completely inhibits germination.


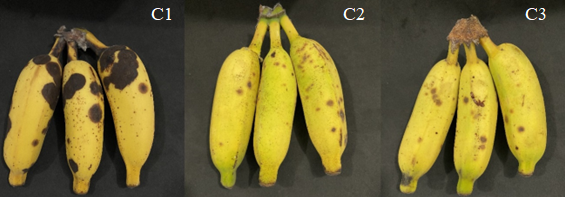


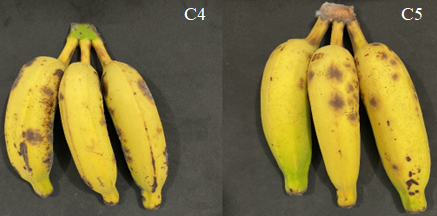


**Figure S10.** Effects of different concentrations of basil essential oil on banana anthracnose disease in in vivo studies.


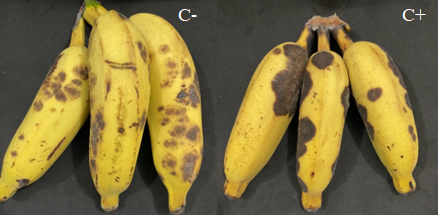


* C1: 0,08; C2: 0,20; C3: 0,40; C4: 0,60 e C5: 0,80 μL mL^-1^. C-: sterile water. C+: Commercial fungicide (0,92 μL mL^-1^).
